# Correction: Metabolic modeling of microbial communities in the chicken ceca reveals a landscape of competition and co-operation

**DOI:** 10.1186/s40168-025-02323-3

**Published:** 2025-12-22

**Authors:** Irina Utkina, Yi Fan, Benjamin P. Willing, John Parkinson

**Affiliations:** 1 Department of Molecular Genetics, University of Toronto, Toronto, Canada; 2 Program in Molecular Medicine, Hospital for Sick Children, Toronto, Canada; 3 Department of Agricultural, Food and Nutritional Science, University of Alberta, Edmonton, Canada; 4 Department of Biochemistry, University of Toronto, Toronto, Canada

**Correction: Microbiome 13, 248 (2025)**

**https://doi.org/10.1186/s40168-025-02241-4**

Following publication of the original article [1], the author reported that Figures 1 and 6 were not the updated version.

The incorrect figures are
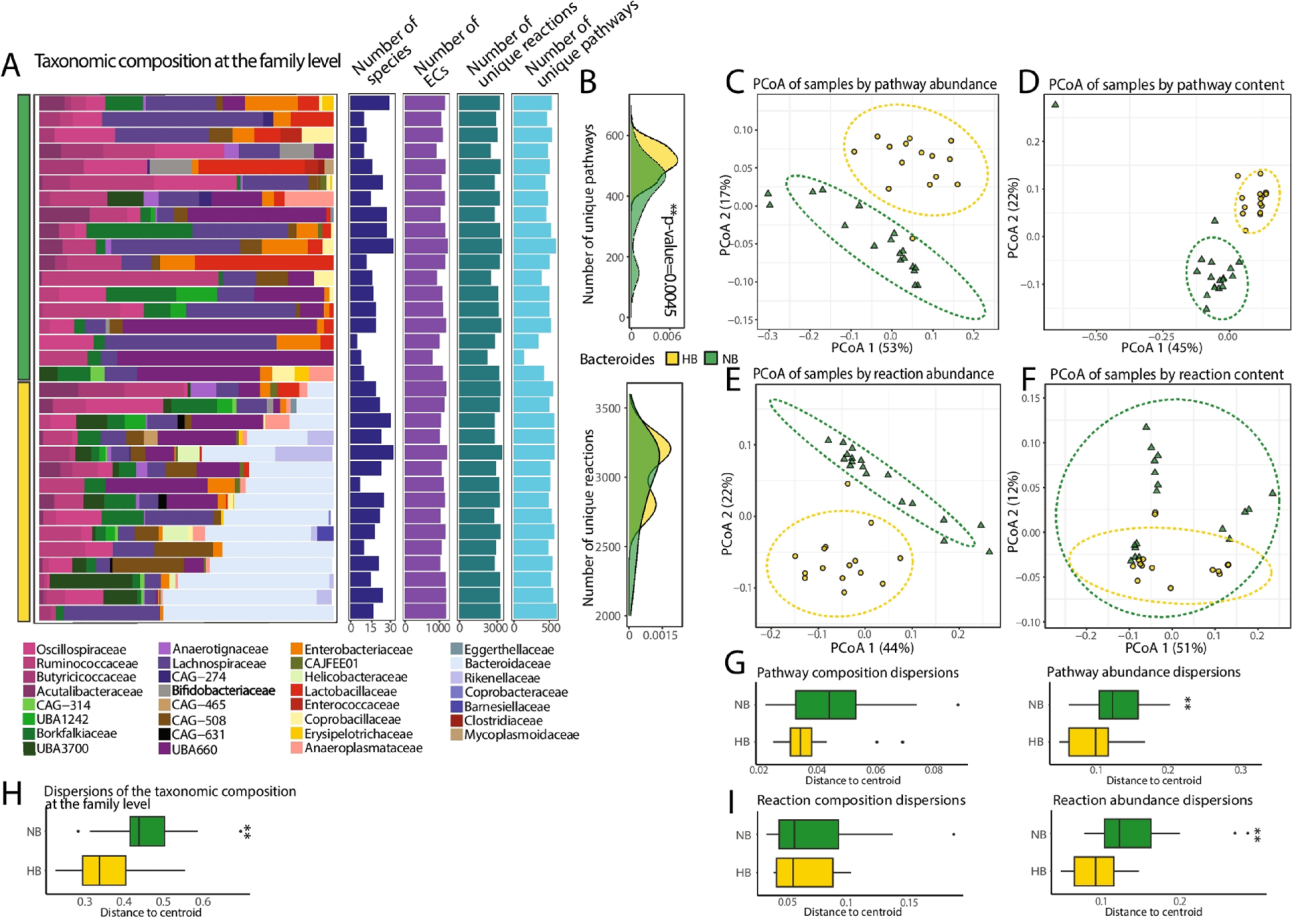



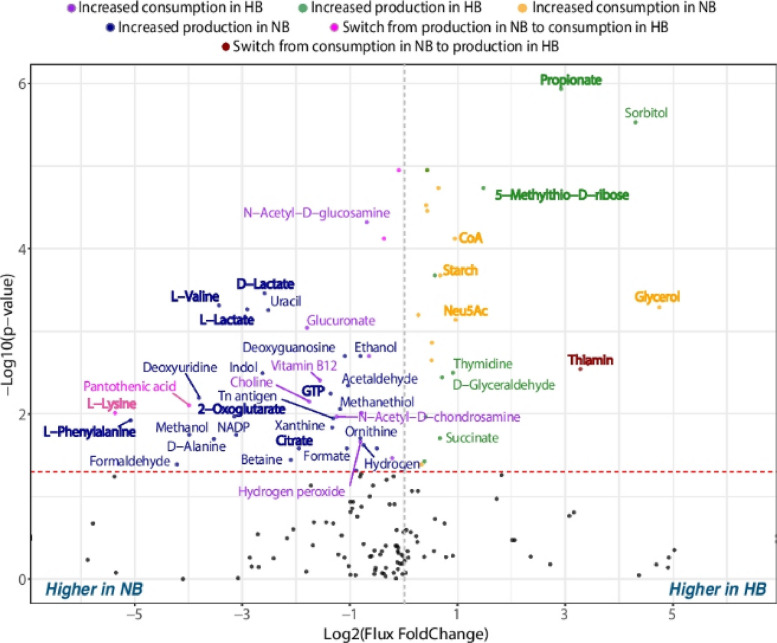


The correct figures are
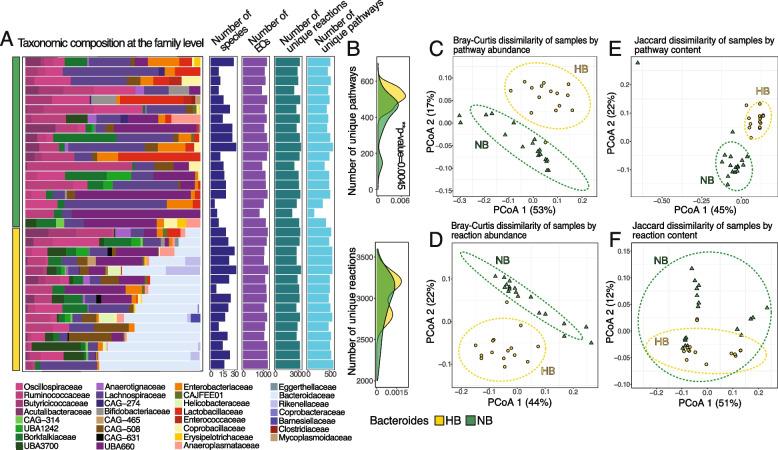



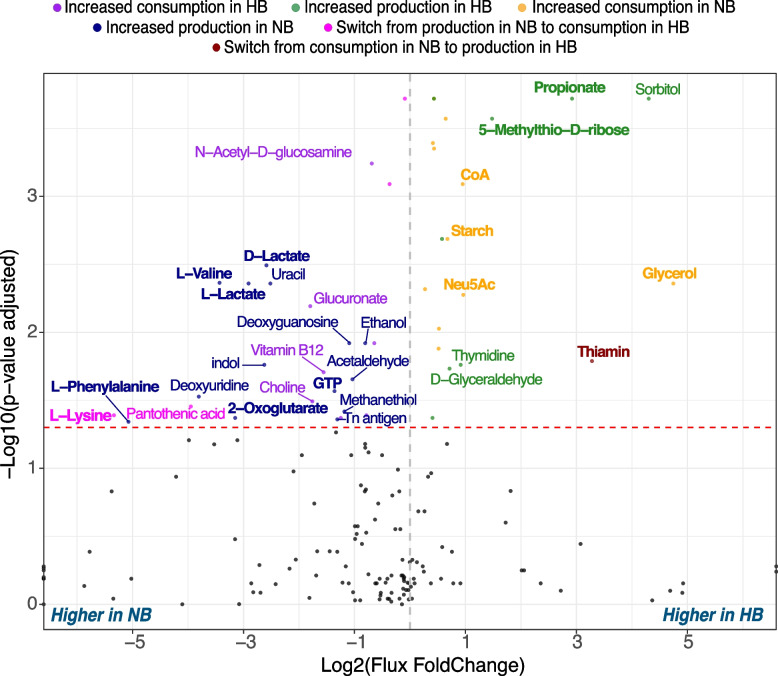


The original article has been updated.
