# Supplementary material for: Metabolic modeling of microbial communities in the chicken ceca reveals a landscape of competition and co-operation
Source: Microbiome. 2025 Nov 27;13:248. doi: 10.1186/s40168-025-02241-4 (PMC12661832; doi:10.1186/s40168-025-02241-4)
Supplement: Supplementary file 2 — Supplementary Material 1. Supplementary Figure 1. Relative abundances of the common taxa across samples. This heatmap illustrates the relative abundances of the top 52 most common taxa across 33 cecal samples, categorized into high Bacteroides (HB, in gold) and no Bacteroides (NB, in green) groups. Taxa present in at least four samples are shown, with the relative abundance represented by the color intensity of each tile. [file 40168_2025_2241_MOESM1_ESM.pdf]

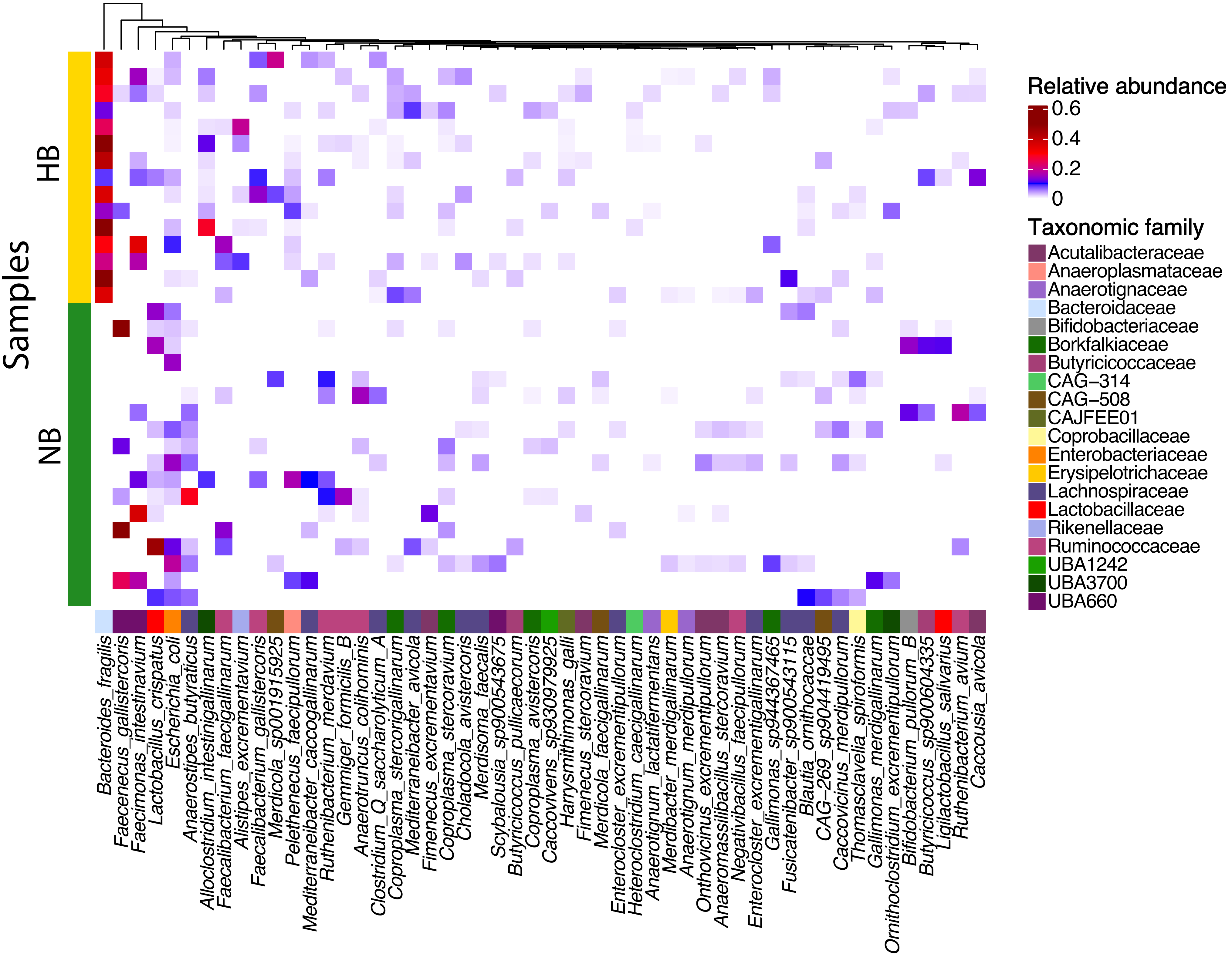

Relative abundance
0.6
0.4
0.2
0
Taxonomic family
Acutalibacteraceae
Anaeroplasmataceae
Anaerotignaceae
Bacteroidaceae
Bifidobacteriaceae
Borkfalkiaceae
Butyricicoccaceae
CAG−314
CAG−508
CAJFEE01
Coprobacillaceae
Enterobacteriaceae
Erysipelotrichaceae
Lachnospiraceae
Lactobacillaceae
Rikenellaceae
Ruminococcaceae
UBA1242
UBA3700
UBA660
Samples
HB
NB
Bacteroides_fragilis
Faecenecus_gallistercoris
Faecimonas_intestinavium
Lactobacillus_crispatus
Escherichia_coli
Anaerostipes_butyraticus
Alloclostridium_intestinigallinarum
Faecalibacterium_faecigallinarum
Alistipes_excrementavium
Faecalibacterium_gallistercoris
Merdicola_sp001915925
Pelethenecus_faecipullorum
Mediterraneibacter_caccogallinarum
Ruthenibacterium_merdavium
Gemmiger_formicilis_B
Anaerotruncus_colihominis
Clostridium_Q_saccharolyticum_A
Coproplasma_stercorigallinarum
Mediterraneibacter_avicola
Fimenecus_excrementavium
Coproplasma_stercoravium
Choladocola_avistercoris
Merdisoma_faecalis
Scybalousia_sp900543675
Butyricicoccus_pullicaecorum
Coproplasma_avistercoris
Caccovivens_sp930979925
Harrysmithimonas_galli
Fimenecus_stercoravium
Merdicola_faecigallinarum
Enterocloster_excrementipullorum
Heteroclostridium_caecigallinarum
Anaerotignum_lactatifermentans
Merdibacter_merdigallinarum
Anaerotignum_merdipullorum
Onthovicinus_excrementipullorum
Anaeromassilibacillus_stercoravium
Negativibacillus_faecipullorum
Enterocloster_excrementigallinarum
Gallimonas_sp944367465
Fusicatenibacter_sp900543115
Blautia_ornithocaccae
CAG−269_sp904419495
Caccovicinus_merdipullorum
Thomasclavelia_spiroformis
Gallimonas_merdigallinarum
Ornithoclostridium_excrementipullorum
Bifidobacterium_pullorum_B
Butyricicoccus_sp900604335
Ligilactobacillus_salivarius
Ruthenibacterium_avium
Caccousia_avicola
