# Supplementary material for: Metabolic modeling of microbial communities in the chicken ceca reveals a landscape of competition and co-operation
Source: Microbiome. 2025 Nov 27;13:248. doi: 10.1186/s40168-025-02241-4 (PMC12661832; doi:10.1186/s40168-025-02241-4)
Supplement: Supplementary file 3 — Supplementary Material 2. Supplementary Figure 2. Distribution of enzyme commission numbers (ECs) across metabolic models. The columns of the heatmap represent individual ECs, while the rows correspond to metabolic models representing different taxa. ECs are annotated using KEGG pathway designations, indicated in two layers to reflect primary and secondary pathway associations. For ECs lacking KEGG pathway annotation, MetaCyc pathway annotation was utilized when available. For ECs mapped to 3 or more major pathway categories, “Multiple superpathways” category was assigned. Each row represents a different taxa, grouped by their presence in HB only, NB only, or both; color-coded. [file 40168_2025_2241_MOESM2_ESM.pdf]

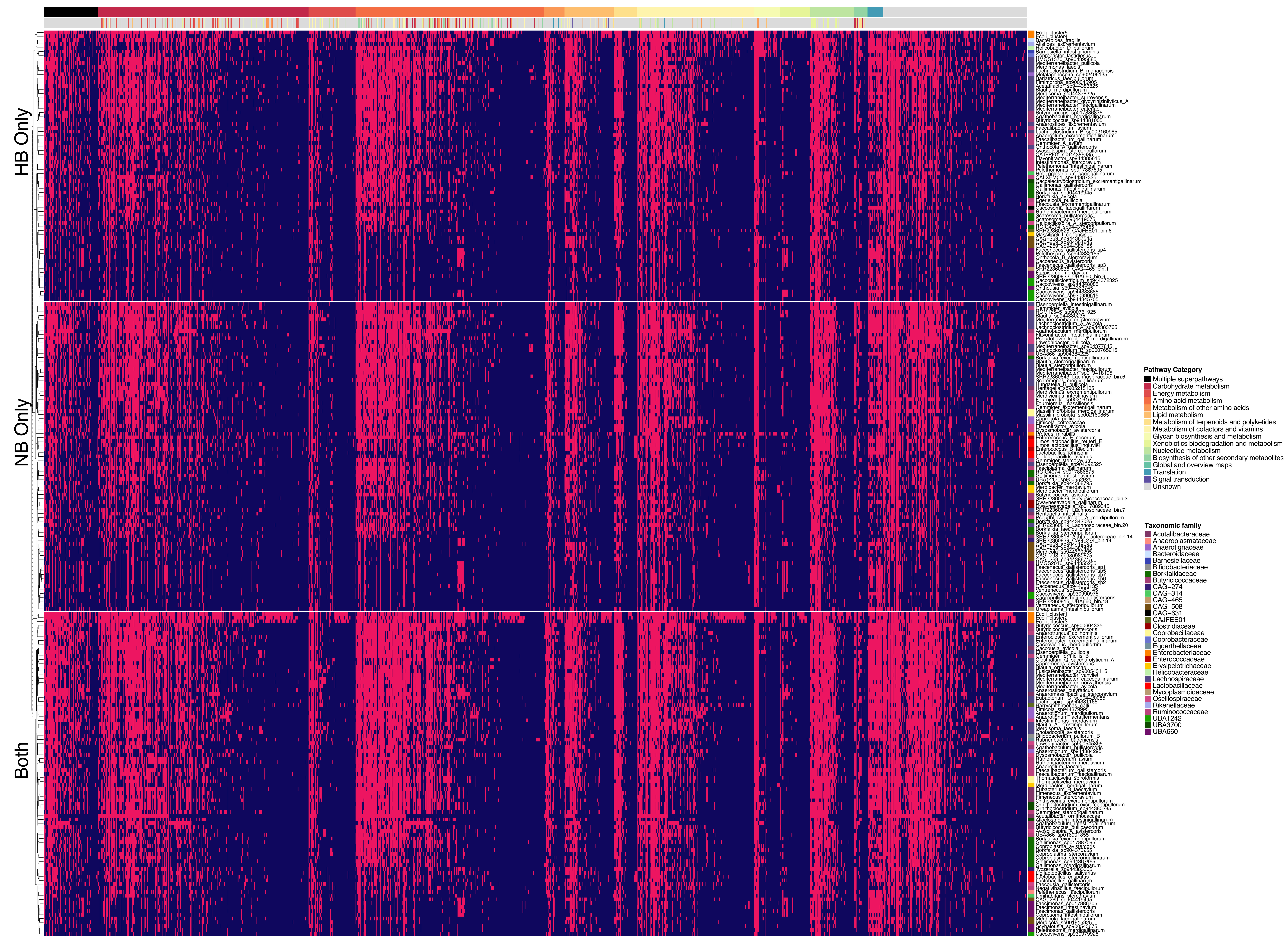
HB Only
NB Only
Both
Pathway Category
Multiple superpathways
Carbohydrate metabolism
Energy metabolism
Amino acid metabolism
Metabolism of other amino acids
Lipid metabolism
Metabolism of terpenoids and polyketides
Metabolism of cofactors and vitamins
Glycan biosynthesis and metabolism
Xenobiotics biodegradation and metabolism
Nucleotide metabolism
Biosynthesis of other secondary metabolites
Global and overview maps
Translation
Signal transduction
Unknown
Taxonomic family
Acutalibacteraceae
Anaeroplasmataceae
Anaerotignaceae
Bacteroidaceae
Barnesiellaceae
Bifidobacteriaceae
Borkfalkiaceae
Butyricicoccaceae
CAG−274
CAG−314
CAG−465
CAG−508
CAG−631
CAJFEE01
Clostridiaceae
Coprobacillaceae
Coprobacteraceae
Eggerthellaceae
Enterobacteriaceae
Enterococcaceae
Erysipelotrichaceae
Helicobacteraceae
Lachnospiraceae
Lactobacillaceae
Mycoplasmoidaceae
Oscillospiraceae
Rikenellaceae
Ruminococcaceae
UBA1242
UBA3700
UBA660
