# Supplementary material for: Metabolic modeling of microbial communities in the chicken ceca reveals a landscape of competition and co-operation
Source: Microbiome. 2025 Nov 27;13:248. doi: 10.1186/s40168-025-02241-4 (PMC12661832; doi:10.1186/s40168-025-02241-4)
Supplement: Supplementary file 5 — Supplementary Material 4. Supplementary Figure 4. SCFA reaction fluxes at the species-level. Cumulative fluxes over 16 hours of simulation for acetate, propionate and butyrate, with samples (x-axis) ordered by the corresponding experimentally measured concentrations – from smallest to largest (from left to right). Colors correspond to the specie contributing to the production or consumption. Positive fluxes correspond to production, while negative fluxes indicated consumption. [file 40168_2025_2241_MOESM4_ESM.pdf]

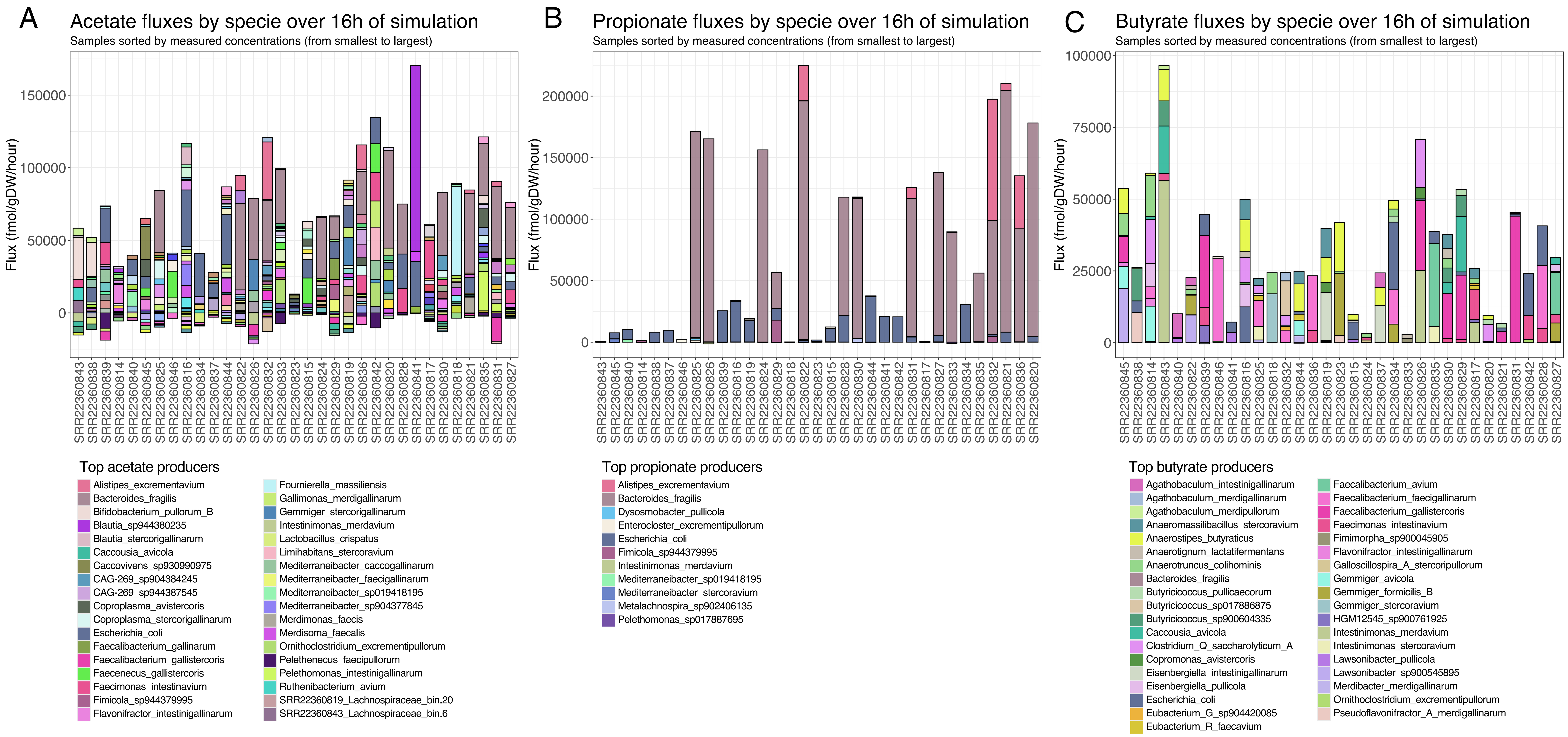
A
Acetate fluxes by specie over 16h of simulation
Samples sorted by measured concentrations (from smallest to largest)
Flux (fmol/gDW/hour)
150000
100000
50000
0
SRR22360843 SRR22360838 SRR22360839 SRR22360814 SRR22360840 SRR22360845 SRR22360825 SRR22360846 SRR22360816 SRR22360834 SRR22360837 SRR22360844 SRR22360822 SRR22360826 SRR22360832 SRR22360833 SRR22360823 SRR22360815 SRR22360824 SRR22360829 SRR22360819 SRR22360836 SRR22360842 SRR22360820 SRR22360828 SRR22360841 SRR22360817 SRR22360830 SRR22360818 SRR22360821 SRR22360835 SRR22360831 SRR22360827
Top acetate producers
Alistipes_excrementavium
Bacteroides_fragilis
Bifidobacterium_pullorum_B
Blautia_sp944380235
Blautia_stercorigallinarum
Caccousia_avicola
Caccovivens_sp930990975
CAG-269_sp904384245
CAG-269_sp944387545
Coproplasma_avistercoris
Coproplasma_stercorigallinarum
Escherichia_coli
Faecalibacterium_gallinarum
Faecalibacterium_gallistercoris
Faecenecus_gallistercoris
Faecimonas_intestinavium
Fimicola_sp944379995
Flavonifractor_intestinigallinarum
Fournierella_massiliensis
Gallimonas_merdigallinarum
Gemmiger_stercorigallinarum
Intestinimonas_merdavium
Lactobacillus_crispatus
Limihabitans_stercoravium
Mediterraneibacter_caccogallinarum
Mediterraneibacter_faecigallinarum
Mediterraneibacter_sp019418195
Mediterraneibacter_sp904377845
Merdimonas_faecis
Merdisoma_faecalis
Ornithoclostridium_excrementipullorum
Pelethenecus_faecipullorum
Pelethomonas_intestinigallinarum
Ruthenibacterium_avium
SRR22360819_Lachnospiraceae_bin.20
SRR22360843_Lachnospiraceae_bin.6
B
Propionate fluxes by specie over 16h of simulation
Samples sorted by measured concentrations (from smallest to largest)
Flux (fmol/gDW/hour)
200000
150000
100000
50000
0
SRR22360843 SRR22360845 SRR22360840 SRR22360814 SRR22360838 SRR22360837 SRR22360846 SRR22360825 SRR22360826 SRR22360839 SRR22360816 SRR22360819 SRR22360824 SRR22360829 SRR22360818 SRR22360822 SRR22360823 SRR22360815 SRR22360828 SRR22360830 SRR22360844 SRR22360841 SRR22360842 SRR22360831 SRR22360817 SRR22360827 SRR22360833 SRR22360834 SRR22360835 SRR22360832 SRR22360821 SRR22360836 SRR22360820
Top propionate producers
Alistipes_excrementavium
Bacteroides_fragilis
Dysosmobacter_pullicola
Enterocloster_excrementipullorum
Escherichia_coli
Fimicola_sp944379995
Intestinimonas_merdavium
Mediterraneibacter_sp019418195
Mediterraneibacter_stercoravium
Metalachnospira_sp902406135
Pelethomonas_sp017887695
C
Butyrate fluxes by specie over 16h of simulation
Samples sorted by measured concentrations (from smallest to largest)
Flux (fmol/gDW/hour)
100000
75000
50000
25000
0
SRR22360845 SRR22360838 SRR22360814 SRR22360843 SRR22360840 SRR22360822 SRR22360839 SRR22360846 SRR22360841 SRR22360816 SRR22360825 SRR22360818 SRR22360832 SRR22360844 SRR22360836 SRR22360819 SRR22360823 SRR22360815 SRR22360824 SRR22360837 SRR22360834 SRR22360833 SRR22360826 SRR22360835 SRR22360829 SRR22360830 SRR22360817 SRR22360820 SRR22360821 SRR22360831 SRR22360842 SRR22360828 SRR22360827
Top butyrate producers
Agathobaculum_intestinigallinarum
Agathobaculum_merdigallinarum
Agathobaculum_merdipullorum
Anaeromassilibacillus_stercoravium
Anaerostipes_butyraticus
Anaerotignum_lactatifermentans
Anaerotruncus_colihominis
Bacteroides_fragilis
Butyricicoccus_pullicaecorum
Butyricicoccus_sp017886875
Butyricicoccus_sp900604335
Caccousia_avicola
Clostridium_Q_saccharolyticum_A
Copromonas_avistercoris
Eisenbergiella_intestinigallinarum
Eisenbergiella_pullicola
Escherichia_coli
Eubacterium_G_sp904420085
Eubacterium_R_faecavium
Faecalibacterium_avium
Faecalibacterium_faecigallinarum
Faecalibacterium_gallistercoris
Faecimonas_intestinavium
Fimimorpha_sp900045905
Flavonifractor_intestinigallinarum
Galloscillospira_A_stercoripullorum
Gemmiger_avicola
Gemmiger_formicilis_B
Gemmiger_stercoravium
HGM12545_sp900761925
Intestinimonas_merdavium
Intestinimonas_stercoravium
Lawsonibacter_pullicola
Lawsonibacter_sp900545895
Merdibacter_merdigallinarum
Ornithoclostridium_excrementipullorum
Pseudoflavonifractor_A_merdigallinarum
