# Supplementary material for: Metabolic modeling of microbial communities in the chicken ceca reveals a landscape of competition and co-operation
Source: Microbiome. 2025 Nov 27;13:248. doi: 10.1186/s40168-025-02241-4 (PMC12661832; doi:10.1186/s40168-025-02241-4)
Supplement: Supplementary file 6 — Supplementary Material 5. Supplementary Figure 5. Influence of species-specific metabolic potential and key taxa presence on cross-feeding activity. A-B. Correlation plots showing the relationship between the average number of unique EC numbers presented in a specie within a sample and (A) the total number of interactions formed by a specie normalized by the number of samples where a specie is present (Pearson) (B) the number of samples where the specie is present (Pearson). Red line is fitted linear regression, and gray area indicates the 95% confidence interval. Each data point represents a species. Only species with at least 10 cross-feeding pairs involving at least 12 metabolites are shown. Selected species (present in at least two samples, above the threshold of 230 total interactions per sample and average number of unique EC per sample > 5) have labels that are color-coded corresponding to the taxonomic family they belong to. C. Effect of E. coli presence across both community categories (HB - High Bacteroides; NB - no Bacteroides) on cross-feeding activity, measured by the cross-feeding coefficient (CFC). Each dot corresponds to a CFC calculated for a single sample. Points and lines are color-coded based on E. coli abundance: red represents communities with high E. coli abundance (>5%), blue represents communities with low E. coli abundance (<5%), and gray represents communities where E. coli is absent. In the NB group, higher E. coli abundance is associated with a notable increase in cross-feeding activity compared to low or absent E. coli, suggesting a significant role for E. coli in enhancing cross-feeding, particularly in the absence of Bacteroides. Conversely, in HB communities, E. coli presence appears to have a more muted effect on cross-feeding, especially in low abundance. D-E. Interaction effects of E. coli and A. butyraticus (D)or L. crispatus (E)on cross-feeding activity, measured by the CFC, across categories. Different colors represent distinct [file 40168_2025_2241_MOESM5_ESM.pdf]

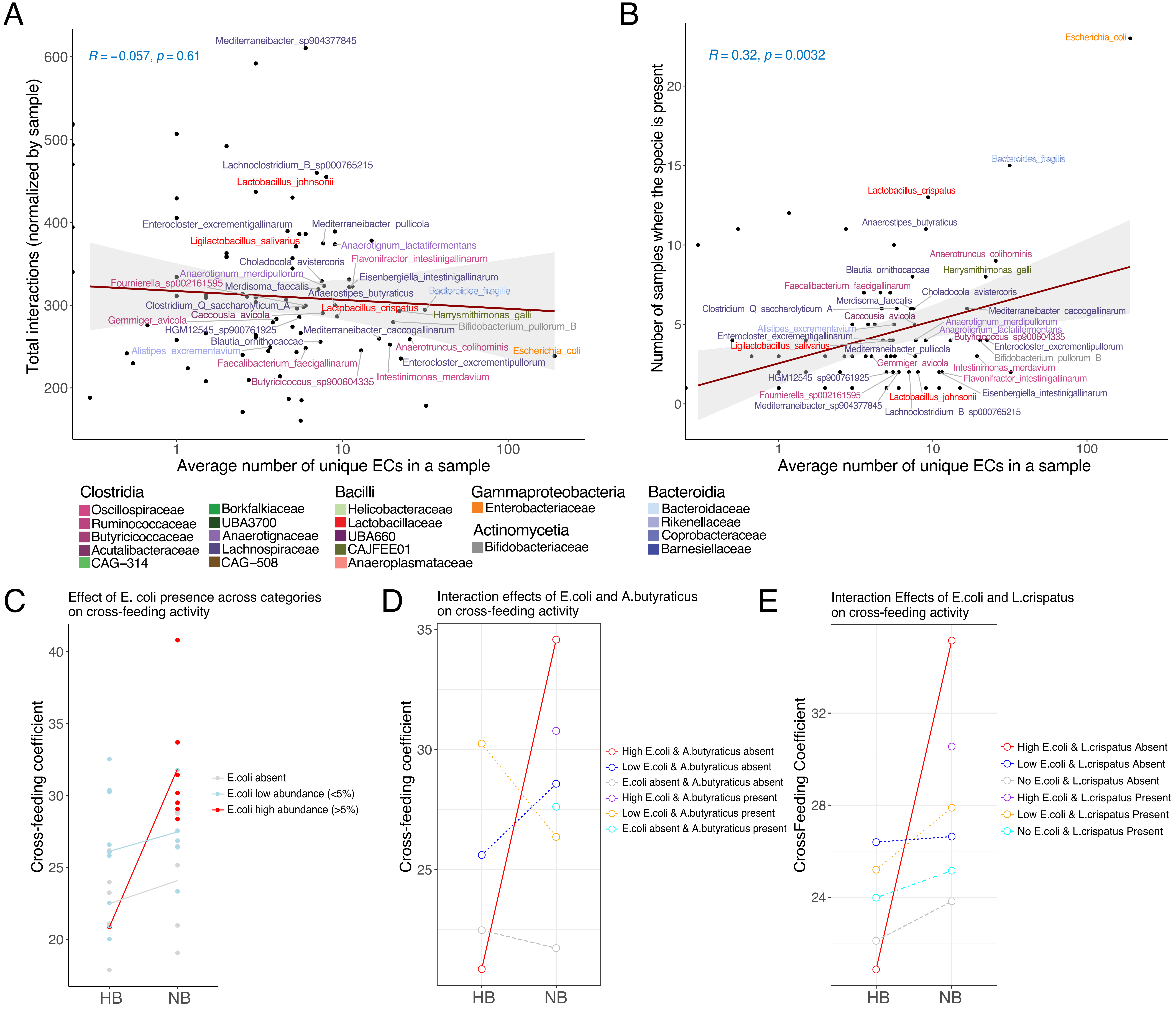

A
R = −0.057, p = 0.61
Total interactions (normalized by sample)
Average number of unique ECs in a sample
Mediterraneibacter_sp904377845
Lachnoclostridium_B_sp000765215
Lactobacillus_johnsonii
Enterocloster_excrementigallinarum
Mediterraneibacter_pullicola
Ligilactobacillus_salivarius
Anaerotignum_lactatifermentans
Choladocola_avistercoris
Flavonifractor_intestinigallinarum
Anaerotignum_merdipullorum
Eisenbergiella_intestinigallinarum
Fournierella_sp002161595
Merdisoma_faecalis
Anaerostipes_butyraticus
Bacteroides_fragilis
Clostridium_Q_saccharolyticum_A
Lactobacillus_crispatus
Caccousia_avicola
Harrysmithimonas_galli
Gemmiger_avicola
HGM12545_sp900761925
Bifidobacterium_pullorum_B
Mediterraneibacter_caccogallinarum
Blautia_ornithocaccae
Alistipes_excrementavium
Anaerotruncus_colihominis
Escherichia_coli
Faecalibacterium_faecigallinarum
Enterocloster_excrementipullorum
Intestinimonas_merdavium
Butyricicoccus_sp900604335
B
R = 0.32, p = 0.0032
Number of samples where the specie is present
Average number of unique ECs in a sample
Clostridia
Oscillospiraceae
Ruminococcaceae
Butyricicoccaceae
Acutalibacteraceae
CAG−314
Borkfalkiaceae
UBA3700
Anaerotignaceae
Lachnospiraceae
CAG−508
Bacilli
Helicobacteraceae
Lactobacillaceae
UBA660
CAJFEE01
Anaeroplasmataceae
Gammaproteobacteria
Enterobacteriaceae
Actinomycetia
Bifidobacteriaceae
Bacteroidia
Bacteroidaceae
Rikenellaceae
Coprobacteraceae
Barnesiellaceae
C
Effect of E. coli presence across categories on cross-feeding activity
Cross-feeding coefficient
E.coli absent
E.coli low abundance (<5%)
E.coli high abundance (>5%)
HB
NB
D
Interaction effects of E.coli and A.butyraticus on cross-feeding activity
Cross-feeding coefficient
High E.coli & A.butyraticus absent
Low E.coli & A.butyraticus absent
E.coli absent & A.butyraticus absent
High E.coli & A.butyraticus present
Low E.coli & A.butyraticus present
E.coli absent & A.butyraticus present
HB
NB
E
Interaction Effects of E.coli and L.crispatus on cross-feeding activity
CrossFeeding Coefficient
High E.coli & L.crispatus Absent
Low E.coli & L.crispatus Absent
No E.coli & L.crispatus Absent
High E.coli & L.crispatus Present
Low E.coli & L.crispatus Present
No E.coli & L.crispatus Present
HB
NB
