# Supplementary figures and images for: Metabolic modeling of microbial communities in the chicken ceca reveals a landscape of competition and co-operation
Source: Microbiome. 2025 Nov 27;13:248. doi: 10.1186/s40168-025-02241-4 (PMC12661832; doi:10.1186/s40168-025-02241-4)

HB Only

**NB**

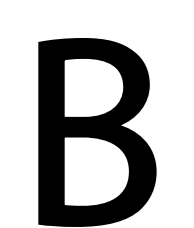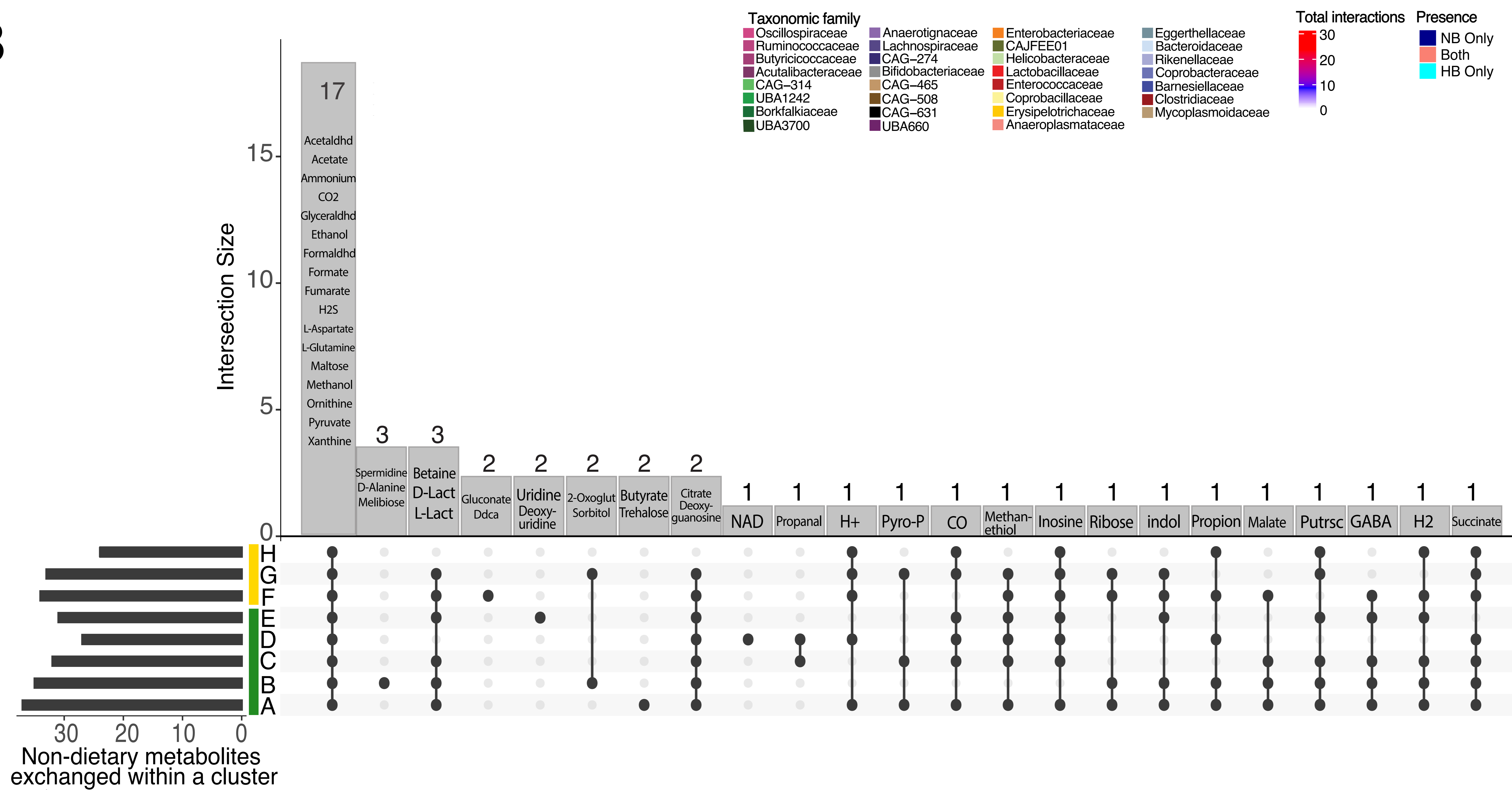

Supplement: Supplementary file 7 — Supplementary Material 6. Supplementary Figure 6. Non-dietary metabolite exchanges across all 227 taxa in HB and NB communities. A. The heatmap displays the normalized total interactions involving non-dietary metabolites exchanged between 227 taxa across 33 chicken cecum samples. Interactions were normalized by the number of samples in which each cross-feeding pair was present and are color-coded by interaction frequency. Taxa are color-coded based on their taxonomic families, interaction frequencies are indicated by color intensity. Taxa on y-axis are grouped by their presence in HB only, NB only, or both; color-coded. Distinct cliques of interacting species are highlighted in red boxes (A-H), which were selected based on visual inspection of the hierarchically clustered heatmap to identify the largest and most distinct clusters of taxa with similar interaction patterns. B. The upset plot summarizes the unique and shared non-dietary metabolites exchanged within the investigated cliques (A-H) highlighted in panel A. Each bar represents the number of metabolites exchanged, with intersections indicating metabolites shared between multiple cliques. Some metabolite names have been shortened or modified for clarity in the figure. The following abbreviations are used: 2-Oxoglut for 2-Oxoglutarate, Ddca for dodecanoic acid, L-Asp for L-Aspartate, Glu for Glutamate, D-Lact and L-Lact for D- and L-Lactate, respectively, NAD for nicotinamide adenosine dinucleotide, Propion for propionate, Pyro-P for pyrophosphate, and Putrsc for putrescine. [file 40168_2025_2241_MOESM6_ESM.pdf]
