# Supplementary material for: Metabolic modeling of microbial communities in the chicken ceca reveals a landscape of competition and co-operation
Source: Microbiome. 2025 Nov 27;13:248. doi: 10.1186/s40168-025-02241-4 (PMC12661832; doi:10.1186/s40168-025-02241-4)
Supplement: Supplementary file 4 — Supplementary Material 3. Supplementary Figure 3. Characteristics of 237 reconstructed MAGs. A-C. Boxplots showing beta-dispersion analysis results - the distances from each sample to the centroid of its respective group (HB or NB). Lower values indicate samples within that group are more similar to each other, while higher values indicate greater within-group variability. The significant differences in dispersion between HB and NB communities (particularly for reaction and pathway abundance) reveal that NB communities exhibit more variable metabolic profiles than HB communities, suggesting that Bacteroides presence may constrain metabolic variability. Distances were calculated from: (A) PCoA based on reaction content (top: Jaccard distances for presence/absence; bottom: Bray-Curtis dissimilarities for abundance-weighted data); (B) PCoA based on pathway content (top: Jaccard distances; bottom: Bray-Curtis dissimilarities); and (C) taxonomic composition at the family level. (). Significance determined using beta-dispersion (TukeyHSD) test. * - p-value < 0.05, ** - p-value <0.01, *** - p-value < 0.001. D. Major characteristics of 237 MAGs reconstructed in this study: completeness, contamination, N50 and genome size. [file 40168_2025_2241_MOESM3_ESM.pdf]

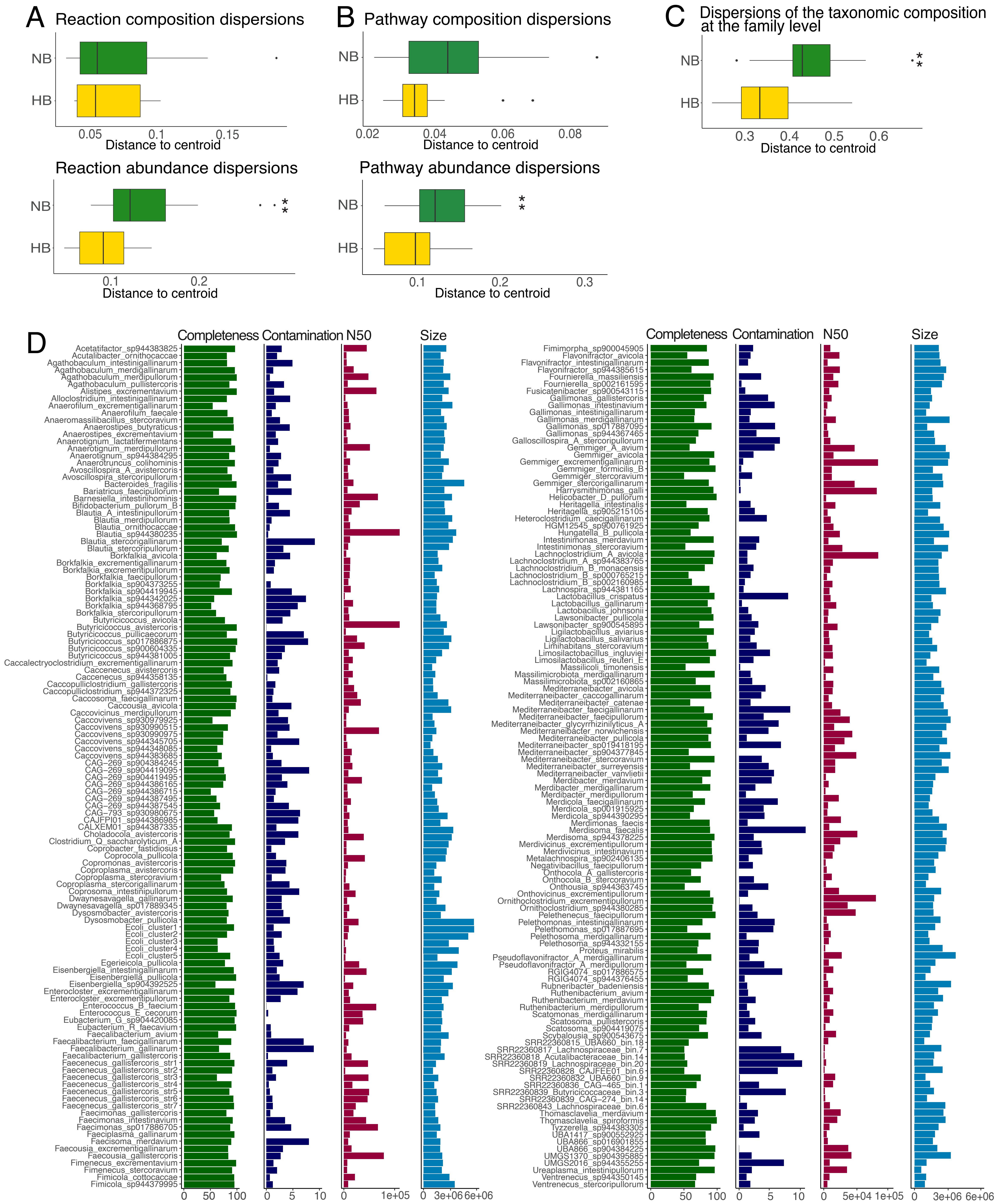

A
Reaction composition dispersions
NB
HB
0.05
0.1
0.15
Distance to centroid
B
Pathway composition dispersions
NB
HB
0.02
0.04
0.06
0.08
Distance to centroid
C
Dispersions of the taxonomic composition at the family level
NB
HB
0.3
0.4
0.5
0.6
Distance to centroid
**
Reaction abundance dispersions
NB
HB
0.1
0.2
Distance to centroid
**
Pathway abundance dispersions
NB
HB
0.1
0.2
0.3
Distance to centroid
**
D
Completeness
Contamination
N50
Size
Acetatifactor_sp944383825
Acutalibacter_ornithocaccae
Agathobaculum_intestinigallinarum
Agathobaculum_merdigallinarum
Agathobaculum_merdipullorum
Agathobaculum_pullistercoris
Alistipes_excrementavium
Alloclostridium_intestinigallinarum
Anaerofilum_excrementigallinarum
Anaerofilum_faecale
Anaeromassilibacillus_stercoravium
Anaerostipes_butyraticus
Anaerostipes_excrementavium
Anaerotignum_lactatifermentans
Anaerotignum_merdipullorum
Anaerotignum_sp944384295
Anaerotruncus_colihominis
Avoscillospira_A_avistercoris
Avoscillospira_stercoripullorum
Bacteroides_fragilis
Bariatricus_faecipullorum
Barnesiella_intestinihominis
Bifidobacterium_pullorum_B
Blautia_A_intestinipullorum
Blautia_merdipullorum
Blautia_ornithocaccae
Blautia_sp944380235
Blautia_stercorigallinarum
Blautia_stercoripullorum
Borkfalkia_avicola
Borkfalkia_excrementigallinarum
Borkfalkia_excrementipullorum
Borkfalkia_faecipullorum
Borkfalkia_sp904373255
Borkfalkia_sp904419945
Borkfalkia_sp944342025
Borkfalkia_sp944368795
Borkfalkia_stercoripullorum
Butyricicoccus_avicola
Butyricicoccus_avistercoris
Butyricicoccus_pullicaecorum
Butyricicoccus_sp017886875
Butyricicoccus_sp900604335
Butyricicoccus_sp944381005
Caccalectryoclostridium_excrementigallinarum
Caccenecus_avistercoris
Caccenecus_sp944358135
Caccopulliclostridium_gallistercoris
Caccopulliclostridium_sp944372325
Caccosoma_faecigallinarum
Caccousia_avicola
Caccovicinus_merdipullorum
Caccovivens_sp930979925
Caccovivens_sp930990515
Caccovivens_sp930990975
Caccovivens_sp944345705
Caccovivens_sp944348085
Caccovivens_sp944383685
CAG-269_sp904384245
CAG-269_sp904419095
CAG-269_sp904419495
CAG-269_sp944386165
CAG-269_sp944386715
CAG-269_sp944387495
CAG-269_sp944387545
CAG-793_sp930980675
CAJFPI01_sp944386985
CALXEM01_sp944387335
Choladocola_avistercoris
Clostridium_Q_saccharolyticum_A
Coprobacter_fastidiosus
Coprocola_pullicola
Copromonas_avistercoris
Coproplasma_avistercoris
Coproplasma_stercoravium
Coproplasma_stercorigallinarum
Coprosoma_intestinipullorum
Dwaynesavagella_gallinarum
Dwaynesavagella_sp017889345
Dysosmobacter_avistercoris
Dysosmobacter_pullicola
Ecoli_cluster1
Ecoli_cluster2
Ecoli_cluster3
Ecoli_cluster4
Ecoli_cluster5
Egerieicola_pullicola
Eisenbergiella_intestinigallinarum
Eisenbergiella_pullicola
Eisenbergiella_sp904392525
Enterocloster_excrementigallinarum
Enterocloster_excrementipullorum
Enterococcus_B_faecium
Enterococcus_E_cecorum
Eubacterium_G_sp904420085
Eubacterium_R_faecavium
Faecalibacterium_avium
Faecalibacterium_faecigallinarum
Faecalibacterium_gallinarum
Faecalicatena_gallistercoris
Faecenecus_gallistercoris_str1
Faecenecus_gallistercoris_str2
Faecenecus_gallistercoris_str3
Faecenecus_gallistercoris_str4
Faecenecus_gallistercoris_str5
Faecenecus_gallistercoris_str6
Faecenecus_gallistercoris_str7
Faecimonas_gallistercoris
Faecimonas_intestinavium
Faecimonas_sp017886705
Faeciplasma_gallinarum
Faecisoma_merdavium
Faecousia_excrementigallinarum
Faecousia_gallistercoris
Fimenecus_excrementavium
Fimenecus_stercoravium
Fimicola_cottocaccae
Fimicola_sp944379995
0
50
100
0
5
10
0
1e+05
0
3e+06
6e+06
Completeness
Contamination
N50
Size
Fimimorpha_sp900045905
Flavonifractor_avicola
Flavonifractor_intestinigallinarum
Flavonifractor_sp944385615
Fournierella_massiliensis
Fournierella_sp002161595
Fusicatenibacter_sp900543115
Gallimonas_gallistercoris
Gallimonas_intestinavium
Gallimonas_intestinigallinarum
Gallimonas_merdigallinarum
Gallimonas_sp017887095
Gallimonas_sp944367465
Galloscillospira_A_stercoripullorum
Gemmiger_A_avium
Gemmiger_avicola
Gemmiger_excrementigallinarum
Gemmiger_formicilis_B
Gemmiger_stercoravium
Gemmiger_stercorigallinarum
Harrysmithimonas_galli
Helicobacter_D_pullorum
Heritagella_intestinalis
Heritagella_sp905215105
Heteroclostridium_caecigallinarum
HGM12545_sp900761925
Hungatella_B_pullicola
Intestinimonas_merdavium
Intestinimonas_stercoravium
Lachnoclostridium_A_avicola
Lachnoclostridium_A_sp944383765
Lachnoclostridium_B_monacensis
Lachnoclostridium_B_sp000765215
Lachnoclostridium_B_sp002160985
Lachnospira_sp944381165
Lactobacillus_crispatus
Lactobacillus_gallinarum
Lactobacillus_johnsonii
Lawsonibacter_pullicola
Lawsonibacter_sp900545895
Ligilactobacillus_aviarius
Ligilactobacillus_salivarius
Limihabitans_stercoravium
Limosilactobacillus_ingluviei
Limosilactobacillus_reuteri_E
Massilicoli_timonensis
Massilimicrobiota_merdigallinarum
Massilimicrobiota_sp002160865
Mediterraneibacter_avicola
Mediterraneibacter_caccogallinarum
Mediterraneibacter_catenae
Mediterraneibacter_faecigallinarum
Mediterraneibacter_faecipullorum
Mediterraneibacter_glycyrrhizinilyticus_A
Mediterraneibacter_norwichensis
Mediterraneibacter_pullicola
Mediterraneibacter_sp019418195
Mediterraneibacter_sp904377845
Mediterraneibacter_stercoravium
Mediterraneibacter_surreyensis
Mediterraneibacter_vanvlietii
Merdibacter_merdavium
Merdibacter_merdigallinarum
Merdibacter_merdipullorum
Merdicola_faecigallinarum
Merdicola_sp001915925
Merdicola_sp944390295
Merdimonas_faecis
Merdisoma_faecalis
Merdisoma_sp944378225
Merdivicinus_excrementipullorum
Merdivicinus_intestinavium
Metalachnospira_sp902406135
Negativibacillus_faecipullorum
Onthocola_A_gallistercoris
Onthocola_B_stercoravium
Onthousia_sp944363745
Onthovicinus_excrementipullorum
Ornithoclostridium_excrementipullorum
Ornithoclostridium_sp944380285
Pelethenecus_faecipullorum
Pelethomonas_intestinigallinarum
Pelethomonas_sp017887695
Pelethosoma_merdigallinarum
Pelethosoma_sp944332155
Proteus_mirabilis
Pseudoflavonifractor_A_merdigallinarum
Pseudoflavonifractor_A_merdipullorum
RGIG4074_sp017886575
RGIG4074_sp944376455
Rubneribacter_badeniensis
Ruthenibacterium_avium
Ruthenibacterium_merdavium
Ruthenibacterium_merdipullorum
Scatomonas_merdigallinarum
Scatosoma_pullistercoris
Scatosoma_sp904419075
Scybalousia_sp900543675
SRR22360815_UBA660_bin.18
SRR22360817_Lachnospiraceae_bin.7
SRR22360818_Acutalibacteraceae_bin.14
SRR22360819_Lachnospiraceae_bin.20
SRR22360828_CAJFEE01_bin.6
SRR22360832_UBA660_bin.9
SRR22360836_CAG-465_bin.1
SRR22360839_Butyricicoccaceae_bin.3
SRR22360839_CAG-274_bin.14
SRR22360843_Lachnospiraceae_bin.6
Thomasclavelia_merdavium
Thomasclavelia_spiroformis
Tyzzerella_sp944383305
UBA1417_sp900552925
UBA866_sp016901855
UBA866_sp904384225
UMGS1370_sp944395885
UMGS2016_sp944355255
Ureaplasma_intestinipullorum
Ventrenecus_sp944350145
Ventrenecus_stercoripullorum
0
50
100
0
5
10
0
5e+04
1e+05
0
3e+06
6e+06
